# Hygiene of Carpets

**Published:** 1890-05

**Authors:** 


### HYGIENE OF CARPETS.

Carpets are commonly made to cover the entire floor of rooms. This cannot be too much deprecated. Carpets, like curtains, are mere dirt traps, which become loaded with filth of every description. This is abundantly proved when a carpet is swept, and the dust allowed to settle on all the articles in the room. Such dust, if examined, will be found to consist of dried mud, chiefly granite or wood, but also containing every description of vegetable and animal impurities. When raised by walking about a room, it is a common cause of "colds" and bronchitis. In addition, as it consists largely of organic matter, it produces a close smell, and contaminates the entering air.

The substitution of a central carpet for one covering the entire floor, is a great improvement, the floor around the carpet being covered with parquet veneering; or, if the expense of this be too great, the whole floor may be painted with four good coats of dark oil paint, and varnished, the joints of the boards having first been made secure. The carpet should be easily removable, in order that it and the floor may be thoroughly cleansed at intervals. Rugs will be found even better, since they may be taken up and shaken every day, if necessary.

In bedrooms, the less carpet the better. Good Chinese or Indian matting is strongly recommended instead, as it does not retain the dust and other impurities which become fixed in the woolly texture of a carpet.—*Good Health.*

